# Comparison of Hypertension in Migrant and Local Patients with Atherosclerotic Diseases: A Cross-Sectional Study in Shanghai, China

**DOI:** 10.5334/aogh.2635

**Published:** 2020-02-28

**Authors:** Xin Gong, Jianwei Shi, Jiaoling Huang, Wenya Yu, Xiaojie Bo, Kangjie Xu, Ning Chen, Qian Liu, Chen Chen, Zhaoxin Wang

**Affiliations:** 1 Shanghai Tenth People’s Hospital affiliated to Tongji University School of Medicine, Shanghai, CN; 2 Shanghai East Hospital affiliated to Tongji University School of Medicine, Shanghai, CN; 3 School of Public Health, Shanghai Jiao Tong University School of Medicine, Shanghai, CN; 4 Tongji University, School of Economics and Management, Shanghai, CN; 5 Shanghai Pengpu New Village Community Health Service Center, Shanghai, CN

## Abstract

**Background:**

Hypertension and its complications represent major health problems worldwide and are distributed differently in different populations. This study aimed to reveal the differences between two populations of patients with hypertension who had atherosclerotic complications: local residents in and migrants to the city of Shanghai, China.

**Methods:**

We conducted a cross-sectional study among hospitalized patients with hypertension age 21–65 years in Pudong District. We compared the characteristics of local and migrant patients with hypertension, and analyzed the distribution and risk factors of atherosclerotic complications between these groups.

**Results:**

The proportion of young and uninsured patients with hypertension was higher among migrant than local participants. The rates of stroke (15.4% vs. 25.0%, p < 0.05) and coronary heart disease (8.6% vs. 11.7%, p < 0.05) were lower and the rates of other atherosclerotic diseases higher (8.5% vs. 7.9%, p = 0.429) among migrant than local participants. According to logistic regression analysis, age was an important risk factor in both the migrant and local groups for all three atherosclerotic complications investigated. Insurance, diabetes, and frequency of hospitalization could influence the incidence of atherosclerotic complications among local patients with hypertension. Among migrant patients, differences for sex, insurance, marital status, diabetes history, and frequency of hospitalization were not significant.

**Conclusions:**

Our study demonstrated differences in the characteristics, distribution, and risk factors of atherosclerotic complications among migrant and local patients with hypertension. Greater attention in needed for the increasing population of migrants.

## Introduction

Hypertension is the main risk factor for atherosclerotic diseases, such as cardiovascular disease [1]. Cardiovascular disease, including coronary heart disease (CHD) and stroke, is one of the leading causes of morbidity and mortality globally [2,3]. In addition to hypertension, atherosclerotic diseases have many other risk factors such as age, sex, and diabetes. At the same time, social determinants should not be ignored in the prevalence of hypertension and its complications. However, social determinants, including residential environment, insurance coverage, and marital status, are even more complex. These can be unequally distributed, even within the same society, and exert different influences at different stages of the life course [4].

As control of hypertension and its risk factors have been highlighted as public health priorities in government health programs worldwide [5,6,7], there are many studies on the prevalence and risk factors of hypertension and its complications, especially atherosclerotic diseases. Most studies have focused on older populations because of the global problem of rapidly aging populations [8]. However, many studies on social determinants tend to research the differences between populations of different ethnicities or different socioeconomic levels [9]. Although the prevention of hypertension is strongly emphasized worldwide, a large discrepancy remains between socioeconomic, regional, and ethnic groups in both developed and developing countries [10,11,12,13], including China [14].

Data from all countries and age groups indicate that in the past four decades, the highest blood pressure levels worldwide have shifted from elderly to middle-aged adults [15,16]. The same applies in China, where among people age 35–75 years, 44.7% have hypertension; of these, 30.1% are being treated but only 7.2% have controlled blood pressure [17]. One specific group, the migrant population, is increasing continuously, owing to the developing economy and improved transportation in China. However, this population has not received sufficient attention in the investigation of risk factors for hypertension. Compared with local patients in the same community, migrant patients with hypertension often have unique traits [18,19]. Most studies of migrant populations in Europe and the United States have focused on the incidence and control rate of hypertension. Compared with the local population, migrants have higher prevalence rates of hypertension and lower rates of awareness and control of hypertension [20,21,22]. However, there are few studies on the complications and risk factors in migrant patients with hypertension, especially in developing countries.

Therefore, we aimed to investigate this problem among middle-aged migrants in Shanghai, China. Shanghai is one of the most developed cities in the country, and many migrants have relocated there from other parts of China [23]. Migrants in Shanghai differ from those in other studies because they are the same ethnicity as that of the local population. Thus, we sought to identify differences between migrant and local populations with respect to: (1) the characteristics of middle-aged patients with hypertension; (2) the proportion of atherosclerotic complications in patients with severe hypertension; (3) the risk factors of atherosclerotic complications in patients with severe hypertension. Our findings may reveal new information for countries with a high number of migrants, to better manage hypertension and atherosclerotic complications in this population.

## Methods

### Study design and data collection

As the largest district of Shanghai, Pudong has a population of 5.47 million, with 2.34 million migrants from other cities residing in Pudong at the end of 2016. The present study was designed as a cross-sectional study according to diagnosis, based on the official discharge database of the Pudong Institute for Health Development. We focused on severe hypertension patients who were hospitalized owing to hypertension from January 1, 2013 to October 31, 2016. We chose 2013 as the start date because the information in the official database was integrated beginning that year. In total, 26 hospitals in Pudong District were covered, whether public or private, specialized or general.

The discharge database for inpatients is composed of three parts. The first part contains the patients’ personal information, including their sex, age, identification card number, birthplace, and native place (in China, native place means the locality where your father was born), profession, present address, and marital status. This information is usually provided by the patient or their family. The second part of the database contains inpatients’ hospitalization information, including diagnosis code, discharge status, pathologic diagnosis (if available), and operation code (if possible). This information is provided by each patient’s physician, which ensures its reliability. Each inpatient is given a diagnosis code by their physician according to disease codes of the International Classification of Diseases 10th Revision (ICD-10). The third part of the inpatient database contains health insurance status (none, registered in the National Health System, or private), and type of hospital (in China, hospitals are classified into three categories according to their main function: community hospitals, secondary hospitals, and tertiary hospitals). This information is automatically generated by the computer system.

Using the information in the database, we were able to assess differences in demographic characteristics between migrants and local patients, and we also extracted information about the diagnosis of hospitalized patients. We collected information on the diagnosis of atherosclerotic diseases, including CHD, stroke, and other atherosclerotic diseases, such as atherosclerosis in lower extremity arteries. We explored the association between atherosclerotic diseases and risk factors in migrants and local patients.

### Study participants

In this study, we selected 24,308 patients whose first diagnosis was hypertension. Consistent with US Joint National Committee and Chinese definitions [24], hypertension was defined as an average systolic blood pressure of at least 140 mmHg or an average diastolic blood pressure of at least 90 mm Hg, or self-reported use of an antihypertensive drug in the past two weeks. Recruited participants were between 21 and 65 years of age and living permanently in Shanghai. Migrants were participants who self-identified as being born in another city and could prove that their parents were born in other cities. Exclusion criteria included pregnant women and those with impaired cognitive ability. We recruited participants age 21–65 years because this age group included the most migrants and we assumed that most participants in this age group would not be in terminal stages of disease. Finally, 6,972 hypertension patients were recruited for this study, including 1,412 migrants.

### Variables

#### Personal characteristics

We included age and sex as demographic variables that may influence hypertension and atherosclerotic complications.

#### Insurance

Insurance is one of the most important factors influencing migrants’ medical behaviors. In China, health insurance includes employment insurance, resident insurance, and commercial insurance. Resident insurance and employment insurance through most companies in one city cannot be used in another city. In our study, we found that very few patients had commercial insurance, so we merged commercial insurance into the category “other.”

#### Marital status

Because the number of widowed, divorced, and single patients was very small, we combined these participants into one group denoted “unmarried.”

#### Diabetes mellitus

Diabetes is considered one of the most important risk factors in atherosclerotic disease, so we included diabetes as a variable. The information about diabetes came from the discharge database, and the diagnosis was based on the ICD-10.

#### Frequency of hospitalization

This variable revealed how many times in total one patient was hospitalized in hospitals throughout Pudong District.

### Statistical analysis

To assess the discrepancy in demographic characteristics between migrants and local residents, we used the chi-square test (Cochran–Mantel–Haenszel test) and t-test. Associations between complications and risk factors were explored using logistic regression analysis. Risk factors included age, sex, marital status, insurance, history of diabetes mellitus, and frequency of hospitalization. All analyses were performed using IBM SPSS, version 20.0 (IBM Corp., Armonk, NY, USA).

## Results

### Characteristics of migrant and local patients

The study population included 6,972 patients age 21–65 years with a primary diagnosis of hypertension. A total of 1,412 participants were migrants and 5,560 were local patients. The proportion of younger patients in the migrant group was significantly larger than that in the local group. Among migrant patients, 11.5% were age 21–35 years, 40.8% were age 36–50 years, and 47.7% were age 51–65 years; among local patients, 78.3% were age 51–65 years. Compared with local patients, fewer migrant patients had insurance (60.8% vs. 7.8% insured, p < 0.05). In addition, fewer migrant patients had diabetes (15.2% vs. 23.7%, p < 0.05) and more migrants were hospitalized for the first time (85.1% vs. 81.1%, p < 0.05). The number of male patients was slightly higher than the number of female patients, with no significant difference between migrant and local patients (p = 0.298). There were no significant differences in marital status between the two groups (93.1% vs. 91.8%, p = 0.098). The main findings for migrant and local participants are reported in Table 1.

**Table 1:** Characteristics of migrant and local participants, n (%).

	Overall (n = 6972)	Migrants (n = 1412)	Local patients (n = 5560)	P value

Age*				0.000
21–35	395(5.7)	163(11.5)	232(4.2)	
36–50	1,549(22.2)	576(40.8)	973(17.5)	
51–65	5,028(72.1)	673(47.7)	4,355(78.3)	
Gender				0.298
Male	3,808(54.6)	859(60.8)	2949(53.0)	
Female	3,164(45.4)	553(39.2)	2611(47.0)	
Marital status				0.098
Married	6,417(92.0)	1315(93.1)	5102(91.8)	
Unmarried (Widowed, divorced, or single)	555(8.0)	97(6.9)	458(8.2)	
Diabetes*				0.000
With	1532(22.0)	215(15.2)	1,317(23.7)	
Without	5,440(78.0)	1,197(84.8)	4,243(76.3)	
Insurance*				0.000
Employment insurance	4,671(67.0)	482(34.1)	4,189(75.3)	
Resident insurance	713(10.2)	47(3.3)	666(12.0)	
Uninsured	1290(18.5)	859(60.8)	431(7.8)	
Other	298(4.3)	24(1.7)	274(4.9)	
Frequency of hospitalization*				0.001
Once	5,712(81.9)	1,201(85.1)	4,511(81.1)	
More than once	1260(18.1)	211(14.9)	1,049(18.9)	

* p < 0.01.

### Distribution of atherosclerotic diseases in migrant and local populations

**Figure 1:**
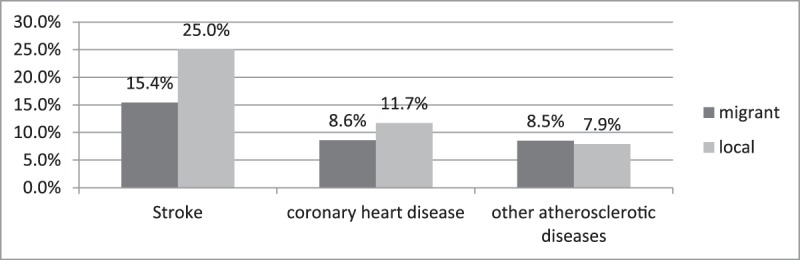
Incidence of atherosclerotic complications in migrant and local groups.

The incidence of the three investigated atherosclerotic diseases in migrant and local populations is presented in Figure 1. The proportion of stroke in migrant inpatients with hypertension was significantly lower than that in local inpatients (15.4% vs. 25.0%, p < 0.05). Similarly, the incidence of CHD among migrant inpatients with hypertension was lower than that in their local counterparts, although not so significantly (8.6% vs. 11.7%, p < 0.05). By comparison, other atherosclerotic diseases occurred more frequently among migrant than local inpatients with hypertension (8.5% vs. 7.9%, p = 0.429).

### Risk factors of atherosclerotic complications in migrant and local populations

In patients with hypertension and history of stroke, migrants had higher rates of patients who were young (21–35 years, 5.0% vs. 1.0%; 36–50 years, 36.2% vs. 11.3%, p < 0.05) and uninsured (61.9% vs. 10.9%, p < 0.05) and showed lower rates of diabetes (17.9% vs. 28.0%, p < 0.05) than local patients. Similarly, in patients with hypertension and a history of CHD and atherosclerotic disease, migrants also demonstrated higher rates of young and uninsured patients, and lower diabetes rates. In particular, in patients with hypertension and a stroke history, more migrant inpatients than local ones were hospitalized for the first time (83.9% vs. 76.9%, p < 0.05). There were more male patients in the migrant than in the local group (74.6% vs. 54.0%, p < 0.05); this was also the case in patients with hypertension and CHD (74.6% vs. 54.0%, p < 0.05). Differences in the risk factors of atherosclerotic diseases among migrant and local populations are reported in Table 2.

**Table 2:** Differences in risk factors of atherosclerotic complications among migrant and local populations.

	Stroke (n = 1610)			Coronary heart disease (n = 772)			Other atherosclerotic diseases (n = 557)		

	Migrant (n = 218)	Local (n = 1392)	P value	Migrant (n = 122)	Local (n = 650)	P value	Migrant (n = 120)	Local (n = 437)	P value

Age			0.000			0.000			0.000
21–35	11(5.0)	14(1.0)		10(8.2)	5(0.8)		6(5.0)	7(1.6)	
36–50	79(36.2)	157(11.3)		25(20.5)	56(8.6)		37(30.8)	60(13.7)	
51–65	128(58.7)	1221(87.1)		87(71.3)	589(90.6)		77(64.2)	370(84.7)	
Gender			0.826			0.000			0.179
Male	137(62.8)	864(62.1)		91(74.6)	351(54.0)		73(60.8)	235(53.8)	
Female	81(37.2)	528(37.9)		31(25.4)	299(46.0)		47(39.2)	202(46.2)	
Marital status			0.626			0.256			0.650
Married	199(91.3)	1251(89.9)		116(95.1)	599(92.2)		115(95.8)	413(94.5)	
Unmarried (Widowed, divorced, or single)	19(8.7)	141(10.1)		6(4.9)	51(7.8)		5(4.2)	24(5.5)	
Diabetes			0.002			0.012			0.020
With	39(17.9)	390(28.0)		29(23.8)	231(35.5)		18(15.0)	111(25.4)	
Without	179(82.1)	1002(72.0)		93(76.2)	419(64.5)		102(85.0)	326(74.6)	
Insurance			0.000			0.000			0.000
Employment insurance	79(36.2)	1043(74.9)		46(37.7)	497(76.5)		30(25.0)	323(73.9)	
Resident insurance	3(1.4)	132(9.5)		2(1.6)	79(12.2)		4(3.3)	73(16.7)	
Uninsured	135(61.9)	152(10.9)		74(60.7)	38(5.8)		86(71.7)	18(4.1)	
Other	1(0.5)	65(4.7)		38(5.8)	36(5.5)		0(0.0)	23(5.3)	
Frequency of hospitalization			0.018			0.734			0.155
Once	183(83.9)	1070(76.9)		86(70.5)	468(72.0)		102(85.0)	344(78.7)	
More than once	35(16.1)	322(23.1)		36(29.5)	182(28.0)		18(15.0)	93(21.3)	

* p < 0.05.

We used a logistic regression model to analyze the differences in risk factors of all atherosclerotic diseases between migrant and local patients with hypertension (Table 3). In the multivariable model of stroke, patients age 51–65 years were found to be at higher risk in both groups. However, the difference in age seemed less apparent in the migrant (odds ratio (OR) = 3.899, p < 0.05) than the local group with hypertension (OR = 7.031, p < 0.05). In addition, uninsured patients (OR = 1.765, p < 0.05), unmarried patients (OR = 1.454, p < 0.05), patients with diabetes (OR = 1.292, p < 0.05), and readmitted patients (OR = 1.391, p < 0.05) were at higher risk of stroke than local patients, and the risk for female participants was lower (OR = 0.580, p < 0.05). However, among migrant patients, differences for sex, insurance, marital status, diabetes history, and frequency of hospitalization were not significant. In the multivariable model of CHD, older patients had a higher risk of CHD in both the migrant (OR = 3.089, p < 0.05) and local groups (OR = 6.530, p < 0.05). Patients who were hospitalized more than once were at higher risk of CHD in both migrant (OR = 2.238, p < 0.05) and local groups (OR = 1.615, p < 0.05). Patients with diabetes had higher risk in the local group only (OR = 1.787, p < 0.05). In the multivariable model of other atherosclerotic diseases, significant differences were found in patients aged 51–65 years between the migrant (OR = 3.353, p < 0.05) and local groups (OR = 2.728, p < 0.05). In addition, uninsured patients showed lower risk of other atherosclerotic diseases in the local group (OR = 0.517, p < 0.05) but this risk was higher in migrants (OR = 1.415, p = 0.151).

**Table 3:** Logistic regression analysis of risk factors in migrant and local patients with hypertension.

Atherosclerotic diseases	Variables		Migrant hypertension patients			Local hypertension patients		

			OR	95%CI	P	OR	95%CI	P

Stroke	Age (year)	21–35	1.000	Reference		1.000	Reference	
		36–50	2.476	1.273–4.814	0.008	3.199	1.809–5.658	0.000
		51–65	3.899	2.010–7.562	0.000	7.031	4.061–12.172	0.000
	Gender	Male	1.000	Reference		1.000	Reference	
		Female	0.827	0.599–1.140	0.246	0.580	0.515–0.665	0.000
	Marital status	Married	1.000	Reference		1.000	Reference	
		Unmarried	1.465	0.840–2.554	0.179	1.454	1.170–1.807	0.001
	Diabetes	Without	1.000	Reference		1.000	Reference	
		With	1.049	0.704–1.565	0.814	1.292	1.121–1.489	0.000
	Insurance	Employment insurance	1.000	Reference		1.000	Reference	
		Resident insurance	0.309	0.093–1.032	0.056	0.733	0.596–0.901	0.003
		Uninsured	0.847	0.602–1.193	0.343	1.765	1.419–2.194	0.000
		Other	0.162	0.021–1.238	0.079	0.973	0.726–1.305	0.857
	Frequency of hospitalization	Once	1.000	Reference		1.000	Reference	
		More than once	1.023	0.675–1.550	0.915	1.391	1.194–1.621	0.000
CHD	Age (year)	21–35	1.000	Reference		1.000	Reference	
		36–50	0.818	0.380–1.763	0.609	2.642	1.044–6.686	0.040
		51–65	3.089	1.512–6.309	0.002	6.530	15.953	0.000
	Gender	Male	1.000	Reference		1.000	Reference	
		Female	0.421	0.267–0.664	0.000	0.869	0.734–1.029	0.104
	Marital status	Married	1.000	Reference		1.000	Reference	
		Unmarried	0.530	0.217–1.292	0.162	1.003	0.735–1.367	0.987
	Diabetes	Without	1.000	Reference		1.000	Reference	
		With	1.165	0.717–1.892	0.537	1.787	1.497–2.132	0.000
	Insurance	Employment insurance	1.000	Reference		1.000	Reference	
		Resident insurance	0.383	0.087–1.678	0.203	0.930	0.719–1.203	0.580
		Uninsured	0.860	0.552–1.339	0.504	0.790	0.555–1.123	0.189
		Other	0.000	0.000–	0.998	1.151	0.796–1.664	0.455
	Frequency of hospitalization	Once	1.000	Reference		1.000	Reference	
		More than once	2.238	1.420–3.527	0.001	1.615	1.335–1.954	0.000
Other atherosclerotic diseases	Age (year)	21–35	1.000	Reference		1.000	Reference	
		36–50	1.803	0.741–4.389	0.194	1.986	0.894–4.411	0.092
		51–65	3.353	1.400–8.028	0.007	2.728	1.272–5.852	0.010
	Gender	Male	1.000	Reference		1.000	Reference	
		Female	0.742	0.493–1.116	0.151	0.877	0.719–1.071	0.197
	Marital status	Married	1.000	Reference		1.000	Reference	
		Unmarried	0.696	0.270–1.794	0.454	0.641	0.419–0.982	0.041
	Diabetes	Without	1.000	Reference		1.000	Reference	
		With	0.835	0.485–1.437	0.514	1.062	0.846–1.332	0.606
	Insurance	Employment insurance	1.000	Reference		1.000	Reference	
		Resident insurance	1.171	0.388–3.533	0.780	1.423	1.086–1.865	0.011
		Uninsured	1.415	0.881–2.272	0.151	0.517	0.318–0.843	0.008
		Other	0.000	0.000–	0.998	1.103	0.708–1.718	0.666
	Frequency of hospitalization	On Once	1.000	Reference		1.000	Reference	
		More than once	1.058	0.613–1.827	0.839	1.095	0.860–1.396	0.461

Abbreviations: OR, odds ratio; CI, confidence interval; CHD, coronary heart disease.

## Discussion

The migrant population in our study had several specific traits. Different from other studies of migrants, the migrant and local groups in our study had the same ethnicity. Nevertheless, we found many differences between migrant and local patients with hypertension. The proportion of young migrant patients with hypertension was significantly higher than in the local group. The higher rate of hypertension in young migrants was consistent with earlier studies [25]. In Italy, Chinese migrant patients with hypertension were 1.4 years younger than local patients, on average [25]. The finding that the rate of diabetes among migrant patients with hypertension was lower than the rate among local hypertensive patients may be owing to the higher proportion of young people in the migrant group.

Compared with local patients, fewer migrants had insurance. In China, all types of resident insurance and most types of employment insurance are not portable from one city to another; therefore, migration would limit the effectiveness of health care among individuals with non-portable health insurance. For example, the non-portable feature of health insurance significantly reduces the likelihood of using medical care (by 7.8%) among rural-to-urban migrants in comparison with rural residents; awareness of hypertension in this migrant population is reduced by 8.8% and the rate of receiving a physician’s advice is reduced by 18.3% [26]. The rate of patients’ being hospitalized for the first time was higher in the migrant group. This may result from the lower rate of insurance among migrants. Improving the portability of health insurance and extending primary care coverage to this population may help to achieve better management of hypertension.

The incidence of hypertension has risen to more than 1.3 billion worldwide. However, the complications of hypertension are not equally distributed worldwide [27]. For different ethnic groups, risk factors associated with cardiovascular disease can be considerably different [28]. Our study revealed that although migrants and local patients were ethnically the same, their complications and risk factors were very different. In our study, we analyzed atherosclerotic complications in these two groups and found that the rates of stroke and CHD were lower among migrants and the rates of other atherosclerotic diseases were higher. When we compared the risk factors of atherosclerotic diseases between migrant and local patients, we found that regardless of whether rates of atherosclerotic complications were lower or higher in the migrant group, risk factor analysis indicated that the rates for young age and first-time hospitalization in migrant patients with hypertension with atherosclerotic complications were much higher than those in their local counterparts. The rates for a history of diabetes and being insured were much lower among migrants with hypertension and atherosclerotic complications.

We found that age was an important risk factor in both migrant and local groups. However, the difference between migrant and local populations was that young people accounted for a larger proportion of migrant patients with hypertension who had atherosclerotic complications. This may suggest that young migrants are under greater pressure to survive and pay less attention to their health; therefore, greater focus on young migrants may be needed.

In migrants with hypertension, diabetes showed no correlation with the three atherosclerotic complications investigated. Diabetes is a commonly known risk factor for atherosclerotic diseases [29,30,31], which is consistent with the results for local patients in our study. This paradox may be related to the lower proportion of diabetes among migrant patients with hypertension. Another possible explanation could be that these migrant patients had a shorter duration of diabetes because of the higher rate of young people among them.

In terms of insurance and frequency of hospitalization, risk factor analysis showed some differences between migrant and local patients with hypertension who had atherosclerotic complications. Among the local group, fewer uninsured patients had milder illnesses or other atherosclerotic diseases. However, more insured patients had relatively serious illnesses such as stroke, suggesting that local uninsured patients tended not to seek medical treatment until an illness became serious. However, among migrants, complications were not affected by the manner of payment, which may be related to the fact that most migrant patients do not have health insurance. In the local population, the number of hospitalizations was not a risk factor for patients with relatively milder illnesses and other atherosclerotic diseases. However, the risk of stroke and CHD in patients with more than one hospitalization was 1.391 and 1.615 times higher than that in patients hospitalized for the first time, respectively, suggesting that patients with severe complications in the local population tended to have been hospitalized in the past. On the contrary, among migrants, the number of hospitalizations was not a risk factor for other atherosclerotic diseases or stroke, suggesting that these patients may not seek medical treatment for the first time until serious disease occurs [32,33,34,35,36]. The lack of insurance makes migrants more likely to experience complications, owing to delayed treatment and to not being hospitalized until complications occur. According to previous studies, uninsured patients are 4.4 times more likely than insured ones to have an unmet need for medical care and prescription drugs [26]. Among uninsured patients, the lack of access to medical care and prescription drugs may not lead to primary diseases such as diabetes but could lead to aggravation or more rapid progression of disease and serious complications. The use of inappropriate drugs, including some OTC medications, may also directly lead to or accelerate complications. Therefore, improving insurance coverage for migrants may help them to have better health-related quality of life.

## Limitations

This study has several limitations. First, although this study covered all hospitals in Pudong District, the area of Shanghai with the largest population of migrants, we did not analyze data from other parts of Shanghai or China. Second, our data were obtained from an official discharge database of Pudong Institute for Health Development, which ensured authenticity and reliability, but limited the comprehensiveness of the data. Data of some possible factors such as smoking and income were not included. Therefore, additional survey and cohort studies are planned as our next steps.

## Conclusion

We found that, compared with local patients who had hypertension, their migrant counterparts were younger and more likely to be uninsured. Thus, greater attention to younger and uninsured migrants is crucial in raising awareness about hypertension and atherosclerotic complications of hypertension. The present study findings indicated that besides age, common risk factors of atherosclerotic disease were not significantly related to atherosclerotic diseases in migrants with hypertension, as compared with their local counterparts. Further research on other social determinants is needed to assess the impact on health in the growing migrant population and to better protect migrants against developing hypertension and complications. Despite its limitations, our study provides good evidence illustrating differences in the characteristics of local and migrant patients with hypertension in China, as well as the distribution and risk factors of atherosclerotic complications. Special attention to the unique migrant population is needed.

## Data Accessibility Statement

The datasets used and analyzed during the current study are available from the corresponding author on reasonable request.

## Abbreviations

CHD: Coronary heart disease

ICD: International Classification of Diseases

OR: odds ratio
